# Supplementary material for: The Systems Biology Research Tool: evolvable open-source software
Source: BMC Syst Biol. 2008 Jun 29;2:55. doi: 10.1186/1752-0509-2-55 (PMC2446383; doi:10.1186/1752-0509-2-55)
Supplement: Additional file 1 — SBRT Archive. An archive of the current version of the Systems Biology Research Tool. [file 1752-0509-2-55-S1.zip › sbrt-1.4.0/doc/developers_guide/api/sbrt/kernel/math/algebra/class-use/DoubleMatrixBuilder.html]

Uses of Interface sbrt.kernel.math.algebra.DoubleMatrixBuilder


|  |  |  |
| --- | --- | --- |
| **Overview** \| **Package** \| **Class** \| **Use** \| **Tree** \| **Deprecated** \| **Index** \| **Help** | |  |
| PREV   NEXT | **FRAMES**    **NO FRAMES**     **All Classes** |  |


---


## **Uses of Interface sbrt.kernel.math.algebra.DoubleMatrixBuilder**

| Packages that use DoubleMatrixBuilder | |
| --- | --- |
| **sbrt.kernel.math.algebra** | Provides classes and interfaces for creating objects relevant to algebra. |

Uses of DoubleMatrixBuilder in sbrt.kernel.math.algebra

| Subinterfaces of DoubleMatrixBuilder in sbrt.kernel.math.algebra | |
| --- | --- |
| `interface` | `SmdMatrix<R,C>`             This interface is used to represent sparse mutable matrices containing strictly double precision numbers as elements. |

| Classes in sbrt.kernel.math.algebra that implement DoubleMatrixBuilder | |
| --- | --- |
| `class` | `AbstractMdMatrix<R,C>`             This abstract class is a skeleton implementation of `DoubleMatrixBuilder`. |
| `class` | `AbstractSmdMatrix<R,C>`             This abstract class is a skeleton implementation of `SmdMatrix`. |
| `class` | `HashMatrix<R,C>`             This class is a `java.util.LinkedHashMap` based implementation of `SmdMatrix`. |
| `class` | `RowOrTreeMatrix<R extends Comparable<? super R>,C extends Comparable<? super C>>`             This class is a `java.util.TreeMap` based implementation of `SmdMatrix`. |
| `class` | `TreeMatrix<R extends Comparable<? super R>,C extends Comparable<? super C>>`             This class is a `java.util.TreeMap` based implementation of `SmdMatrix`. |

Methods in sbrt.kernel.math.algebra with parameters of type DoubleMatrixBuilder

`<R,C,T> void`

`MatrixMultiplier.multiply(SparseDoubleMatrix<R,C> matrixA,
SparseDoubleMatrix<C,T> matrixB,
DoubleMatrixBuilder<R,T> product)`
  
          Fills `product` with `matrixA` \* `matrixB`.

---


|  |  |  |
| --- | --- | --- |
| **Overview** \| **Package** \| **Class** \| **Use** \| **Tree** \| **Deprecated** \| **Index** \| **Help** | |  |
| PREV   NEXT | **FRAMES**    **NO FRAMES**     **All Classes** |  |


---
